# Acceptability of oral rapid HIV testing at dental clinics in communities with high HIV prevalence in South Florida

**DOI:** 10.1371/journal.pone.0196323

**Published:** 2018-04-27

**Authors:** Erin L. P. Bradley, Denise C. Vidot, Zaneta Gaul, Madeline Y. Sutton, Margaret Pereyra

**Affiliations:** 1 Division of HIV/AIDS Prevention, National Centers for HIV, Viral Hepatitis, STD and TB Prevention, Centers for Disease Control and Prevention, Atlanta, GA, United States of America; 2 School of Nursing and Health Studies, University of Miami, Miami, FL, United States of America; 3 ICF, Atlanta, GA, United States of America; 4 Sociomedical Sciences at the Columbia University Medical Center, Columbia University, New York, NY, United States of America; University of Washington, UNITED STATES

## Abstract

**Background:**

Expanding HIV screening for populations at risk necessitates testing in nontraditional settings. We assessed HIV testing in dental clinics in South Florida, an urban area with the highest rates of HIV diagnoses in the United States in 2015.

**Aims:**

We explored patients’ acceptance of oral HIV rapid tests administered by dental providers and identified reasons for accepting or declining HIV testing.

**Methods:**

During 2014 and 2015, dentists and hygienists at two federally qualified health center (FQHC) dental clinics who serve racial/ethnic minority patient populations in South Florida were trained to administer oral HIV rapid tests as a part of a routine dental visit. Patients presenting for dental services were offered a rapid HIV test and brief survey regarding their demographics, HIV testing history and behaviors.

**Results:**

We enrolled 600 patients (median age = 43 years; IQR: 29–56 years), 45% non-Hispanic black and 35% Hispanic/Latino, 83% graduated high school, and 50% unemployed. Most (85%) accepted oral HIV rapid testing (none tested HIV-positive); 14% had never been tested for HIV. The most common reasons for testing were a desire to know HIV status (56%) and free testing (54%). Among 93 (15%) patients who declined testing, 58% were tested recently and 31% felt confident that they were HIV-negative; however, 74 (80%) who declined testing said they would feel comfortable discussing HIV prevention with their dentist. Additionally, 290 of 600 patients (48%) reported condomless vaginal or anal sex in the past 6 months. Further, among 119 patients who had condomless sex with an HIV-positive partner and/or one whose HIV status was unknown, 98 (82%) accepted the oral HIV test.

**Conclusion:**

Dental clinics may provide expanded opportunities for oral HIV rapid testing and conversations about HIV prevention in high HIV prevalence communities.

## Introduction

African American/black and Hispanic/Latino communities are heavily affected by HIV in the United States. Rates of new HIV diagnoses in 2015 for Hispanics/Latinos and African Americans/blacks (referred to hereafter as blacks) were 3 and 8 times that of whites, respectively [1]. At the end of 2014, 63% of the people living with HIV in the United States were black or Hispanic/Latino [1]. The southern United States is also disproportionately affected by HIV. In 2015, the rate of new HIV diagnoses was higher in the South than other regions of the country (16.8 per 100,000 compared to 11.6, 9.8, and 7.6 per 100,000 in the Northeast, West, and Midwest, respectively) [1]. The metropolitan statistical area that includes Miami, Fort Lauderdale, and West Palm Beach, Florida led the country in rates of new HIV diagnoses in 2015 [1]. Miami-Dade County and Broward County, both of which have large black and Hispanic/Latino populations, ranked first and second in Florida for rates of new HIV diagnoses (51.2 and 34.8 per 100,000, respectively) [1].

Because of significant advances in HIV treatment and care, people living with HIV (PLWH) now have the potential to live longer healthier lives [2]. Individuals who are diagnosed early, successfully linked to and retained in care, and able to adhere to antiretroviral therapy (ART) are most likely to achieve and sustain viral suppression [3]. In addition to improved health and quality of life for PLWH, viral suppression supports prevention efforts because individuals who are virally suppressed are less likely to transmit HIV to others [4]. An estimated 30% of new HIV infections may be attributed to undiagnosed individuals transmitting to their partners [5]. Therefore, several public health agencies, including the Centers for Disease Control and Prevention (CDC), have emphasized the importance of individuals being tested for HIV to know their status, especially for communities that are disproportionately affected by HIV [6].

Conducting HIV rapid testing in nontraditional settings may bolster efforts to expand HIV screening for communities at risk for infection by making testing more accessible [7–9]. Since the 2006 release of CDC’s revised HIV testing recommendations that encouraged routine HIV screening in all healthcare settings for adults and adolescents ages 13 to 64 years [10], HIV screening in the dental setting has garnered increased attention. Some have argued that the dental setting is an important but underutilized venue in which HIV rapid testing could be implemented [11–15]. Pollack and colleagues [13] used data from the 2005 National Health Interview Survey to highlight potential missed opportunities for identifying HIV-infected persons who visit the dentist but may not receive care in other healthcare settings where HIV tests are typically offered. However, Silveira and Chattopadhyay [16] questioned the strength of the evidence for dental clinics providing increased access to medical screenings, citing limited access to dental care for economically disadvantaged individuals and only modest potential gains in regards to reach.

Previous studies provide dental professionals’ and patients’ views of HIV rapid testing. Although many dental professionals understand the rationale for oral fluid HIV rapid testing, there is not yet consensus about whether it falls within the scope of dental practice [12, 17–19]. Additionally, common concerns about integrating HIV screening into practice include patient receptivity, inadequate HIV knowledge and training to address counseling, patient confidentiality, and referrals for care, as well as logistics concerns including space, time, staff, or cost/reimbursement [12,18–22]. Therefore, many dental practices may be hesitant to offer HIV testing, but still may be informed by studies that demonstrate interest and feasibility by both patients and dental providers.

Research suggests patients may be more receptive to HIV rapid testing than some providers presume. Studies conducted at a free dental clinic in Kansas City, Missouri [23] and two dental schools in Los Angeles [24] and New York [25] revealed the majority of patients held positive views and would be willing to be tested during dental visits. Reasons for potentially declining were low perceived risk for HIV infection, having already been tested, fear of testing positive, anticipating HIV stigma if positive, and not wanting to extend the length of their visit [23–25]. In one study, patients expressed concerns regarding confidentiality and linkage to HIV care (in the event of a positive HIV test) that were similar to those expressed by dentists in other studies [25]. Findings from the small body of research in this area suggests dental visits may provide a valuable opportunity for HIV testing when dental professionals are adequately trained and protocols to address privacy and logistics concerns are in place.

Currently, there is a dearth of investigations and empirical data about the extent to which HIV rapid testing can be integrated into dental practice and utilized by patients in areas heavily affected by HIV. A search of the peer-reviewed literature yielded only two studies evaluating the uptake of HIV rapid testing in dental settings in the U.S. [26,27], and both took place in the northeastern U.S. Therefore, we sought to examine the acceptability of HIV testing in two dental clinics that primarily serve racial/ethnic minority populations in Miami-Dade and Broward Counties in Florida, two southeastern counties that are heavily affected by HIV infection. We explored patients’ acceptance of oral HIV rapid tests administered in this setting and identified reasons for accepting or declining HIV testing.

## Methods

### Design and setting

For this cross-sectional study, two federally qualified community health care centers (FQHCs) with dental clinics served as recruitment sites from July 2014 to December 2015 to obtain a sample of 600 patients. Both FQHCs provided dental care and primary medical care services on site, including primary care specifically for HIV-positive patients. Both dental clinics had patient populations that represented the groups that are most at risk for HIV in Miami-Dade and Broward Counties, Florida, and nationally: blacks, Hispanics/Latinos, and men who have sex with men. Neither FQHC offered HIV testing in their dental clinics prior to the commencement of this study.

### Participants

Patients receiving dental services at these FQHCs were invited to participate in the research study. Inclusion criteria were: (1) 18 years of age or older; (2) completed a dental visit on the day of recruitment; (3) self-reported that a rapid oral HIV test was offered during the visit; (4) capable of communicating in English, Spanish, or Creole. Patients were excluded if there was evidence of an acute severe psychiatric condition (e.g., active psychosis, manic-depressive illness, imminent suicide) or a self-report of HIV-positive status.

### Procedures

During 2014, dental care providers at these FQHCs were trained to administer OraQuick *ADVANCE*^*®*^ HIV rapid tests (sensitivity ~ 99.3%9; specificity ~ 99.8%)[28] as a part of a routine dental visit. This oral fluid rapid test was used in this study due to the research setting (oral care in dental settings) and the ease of specimen collection for both participants and staff. Rapid test results were available in about 20 minutes. The rapid test training consisted of multiple sessions hosted by an OraQuick *ADVANCE*^*®*^ HIV rapid test trained distributor who demonstrated proper sample collection procedures. After being trained by the distributor, dental care providers attended another training session led by the principal investigator (MP) and project coordinator (DV). This session was dedicated to reviewing the detailed procedures and testing the questionnaire that would be administered to patients by the research staff.

A systematic random sample of 300 patients at each dental location was obtained by inviting every third patient to participate in the study until the desired sample size of 600 persons was reached. This random sampling technique limited the potential for selection bias. Research staff obtained a daily list of patients from the administrative assistant at each clinic to identify every third patient. The dentist or dental hygienist (2 dentists at clinic 1; 2 dentists and 2 dental hygienists at clinic 2) offered the patient the opportunity to receive a rapid oral HIV test during their dental visit and to participate in a survey after their dental visit. The patient responded “yes” or “no” to receiving the rapid oral HIV test and/or survey. If the patient agreed, a trained research staff member approached the patient to screen them for eligibility and obtain written informed consent. During the consent process, the dentist or dental hygienist stepped out to see another patient or to get materials ready for the patient’s dental visit. The consented patients who agreed to the rapid test had an oral swab obtained by the dentist or hygienist during the dental visit, and received their results at the end of the dental visit. Whether accepting or declining testing, all study participants completed the survey in their language of choice (English, Spanish, or Creole) after their visit in a private room using audio computer-assisted self-interview (ACASI) technology to assure confidentiality and enhance accuracy of their responses to sensitive questions, and to address potential literacy issues [29].

At least one research staff member was at the dental clinic during operating hours. The research staff only interfaced with the patient after a decision was made to accept or decline the rapid test from their provider. Research staff followed a recruitment script and received intensive training by the principal investigator and project coordinator on how to approach and engage potential participants. All potential participants were informed that participation in the study activities was voluntary and would not interfere with their dental care if they declined participation. Study participants were provided a small token of appreciation ($25 gift card from a local retailer) after completing the survey. All study protocols were approved by Columbia University’s Institutional Review Board and CDC’s review and project determination process.

### Measures

The questionnaire included items relating to: (1) timing and location of prior HIV tests, (2) reasons for accepting/declining rapid HIV test during the dental visit, (3) sexual risk behavior, and (4) participant demographics. Details of the measures used are outlined below.

#### HIV testing history

At the end of the dental visit, patients were surveyed and asked whether rapid HIV testing was offered by the dentist or the dentist’s ancillary staff (e.g., dental hygienist and/or dental assistant) during that day’s visit. A history of HIV testing and receipt of results within the past year was also determined. If not tested in the past year, patients were asked if they have ever been tested for HIV. All participants who self-reported being HIV-tested at any time were asked to identify the corresponding testing venue for their most recent test.

#### Reasons for accepting or declining HIV testing

Patients also provided reasons for accepting or declining the rapid HIV test during their visit using items developed based on programs developed through the National Association of Community Health Centers’ Model to integrate routine HIV screening services in FQHCs with dental clinics and previously published studies [30,31]. Individuals who accepted the test selected 1 of 5 options as their main reason: (1) importance of knowing HIV status, (2) free testing, (3) rapid results (20-min), (4) comfort testing at the dentist’s office instead of another setting, or (5) other reason. Patients provided additional reasons by indicating “yes” or “no” to a range of options, including belief that they were at risk for HIV, not having prior HIV testing, and trusting the dentist/hygienist (see S1 Appendix for select survey items). Individuals who declined rapid testing during their visit also responded “yes” or “no” to a range of options, including lack of time, embarrassment, fear of knowing their HIV status, and having been tested for HIV recently. Additionally, patients who indicated they had never been tested for HIV reported reasons for not receiving any type of HIV test in the past.

#### HIV/AIDS knowledge

HIV knowledge was assessed using the HIV Knowledge Questionnaire 18-Item (Brief) Version [32]. The questionnaire used dichotomous (true/false) responses to various statements concerning prevention and transmission of HIV (S1 Appendix). Participants received one point for each correct response. Potential scores ranged from 0 to 18, with higher scores indicating more knowledge.

#### Sexual behaviors

Five self-developed items measured the following in the 6 months prior to the interview: total number of vaginal and/or anal sex partners; any unprotected vaginal and/or anal sex; and HIV status of partners with whom they had sex without a condom (positive, negative, or unknown).

#### Demographics

Demographic information, including age, gender, race and ethnicity, years of formal education, income, employment status, and health insurance, was collected. Patients were also asked how long they had been in care with their dentist and to rate their overall general health and oral health.

#### Data analyses

The primary aim of the study was to examine the acceptability of oral HIV rapid testing in a dental setting by determining the percent of patients who consented to an oral HIV rapid test during their regular dental clinic visit. To better understand testing behavior in a dental setting, the secondary aim was to identify reasons for accepting or declining HIV testing.

SPSS 21 was used to generate statistical summaries of data. Medians, interquartile ranges, frequencies, and crosstabs were used to describe demographic characteristics, sexual risk behaviors, and testing acceptance rates and reasons for accepting or declining the oral HIV rapid test.

## Results

### Demographic summary

Of a total of 600 patients enrolled, 45% were non-Hispanic black and 35% were Hispanic/Latino. The median age was 43 years (IQR: 29–56 years). Most (83%) graduated from high school, 50% were unemployed, 41% had an annual household income (past 12 months) less than $10,000, 48% had some form of health insurance, and 46% had dental insurance. Sixty-one percent reported having a spouse or partner. The majority (54%) of patients reported having one sex partner in the past 6 months. The median HIV knowledge score on the HIV Knowledge Questionnaire 18-Item (Brief) Version was 79% correct (IQR: 63–89). Demographic characteristics are provided in Table 1.

**Table 1:** Demographic characteristics for patients receiving dental care at 2 federally qualified healthcare centers, South Florida, 2014–2015 (*N* = 600).

Demographic Characteristics	N (%)
**Race/Ethnicity^a^**	
Hispanic or Latino	209 (34.9)
Non-Hispanic black	272 (45.4)
Non-Hispanic white	102 (17.0)
Other	16 (2.7)
**Gender^b^**	
Female	394 (65.7)
Male	202 (33.7)
Transgender	4 (0.7)
**Employment^a^^,^^b^**	
Full-time	169 (28.2)
Part-time	132 (22.0)
Unemployed	298 (49.7)
**Education^a^**	
Less than high school	103 (17.2)
High school or GED	215 (35.9)
Some college	172 (28.7)
College or graduate degree	109 (18.2)
**Annual Household Income^c^**	
$10,000 or less	203 (40.5)
$10,001 to 20,000	143 (28.5)
$20,001 to 30,000	92 (18.4)
$30,001 or more	63 (12.6)
**Insurance Coverage**	
Health insurance	288 (48.0)
Dental insurance	277 (46.2)
**Relationship Status**	
Have spouse or partner	365 (60.8)
Living with spouse or partner	233 (63.8)^d^
**Age, Mean (SD)**^a^	43 (15.9)
**Sexual Risk Behavior**		
**Condomless vaginal and/or anal sex (6 months)**^a^		289 (48.2)
**Number of sex partners (6 months)**^e^	
0	176 (29.5)
1	319 (53.5)
2 or more	101 (17.0)

Notes

^a^Sample size differed due to missing responses (*N* = 599).

^b^Percentages may not add up to 100 due to rounding.

^c^Sample size differed due to missing responses (*N* = 501).

^d^Calculated based on those who reported having a spouse or partner (*N* = 365).

^e^Sample size differed due to missing responses (*N* = 596).

### Oral rapid HIV test acceptance

Most (85%; n = 507) of the 600 patients surveyed accepted the oral HIV rapid test offered by the dentist or hygienist. Fourteen percent of those accepting the test had never been tested for HIV. The most common reasons for testing among all patients were a desire to know their HIV status (56%; n = 282) and receiving the test for free (54%; n = 272); 24% (n = 121) also indicated that it had been a long time (not specified) since their last HIV test. None tested positive for HIV.

Among the 93 patients who declined testing and reported one or more reasons for doing so, 58% (n = 54) had been tested recently; 31% (n = 29) did not desire to be tested because they felt confident that they were HIV-negative. Another reason for not being tested included not having enough time to wait for their test results (14%; n = 13). Only one patient reported not wanting to be tested in a dental clinic. However, 80% (n = 74) of patients who declined testing said they would feel comfortable discussing HIV prevention with their dentist.

### Self-reported sexual risk and HIV testing

Nearly half (48%; n = 290) of the sample reported having had vaginal or anal sex without a condom in the past 6 months; 60% (n = 171) of those who engaged in sex without a condom reported doing so only with a partner they believed was HIV-negative. Of the remaining 40% who reported condomless sex with a partner who was HIV-positive (n = 30), a partner whose HIV status was unknown (n = 67), or did not report the HIV status of their partner (n = 22), 82% (n = 98) were tested during their dental visit. HIV knowledge and reasons for declining testing did not differ from those reported for the entire sample.

Seventeen percent (n = 101) of the sample reported having two or more sex partners in the past 6 months; 86% (n = 87) of these individuals accepted the oral HIV rapid test. Reasons for declining the test were similar to those for the entire sample, except none endorsed not having enough time. HIV knowledge was slightly higher (Mdn = 84% correct; IQR: 68–95%) compared to the entire sample (Mdn = 79% correct; IQR: 63–89).

## Discussion

Innovative strategies in HIV screening are needed to reach priority populations with known effective HIV prevention services. Findings from our study were consistent with previous research that suggests dental clinic settings may expand opportunities for oral HIV rapid testing and conversations about HIV prevention [23–27]. In this high HIV prevalence area in South Florida, testing was widely accepted by our sample receiving dental services at FQHCs. The 85% acceptance rate for this study was similar to Nassry and colleagues’[27] 88% testing rate when oral HIV rapid testing was offered by a dental faculty member or student in a New York dental school setting, as well as Blackstock and colleagues’[26] 98% acceptance rate among a mostly minority, hospital-based clinic sample in New York City. Findings from these three studies suggest patients are generally open to oral HIV rapid testing in dental settings when offered by a staff member.

The most common reasons for declining a rapid HIV test included previous recent testing, being confident they were HIV-negative, or not having time during their dental visit, which was consistent with previous studies [23–25]. However, even among those who declined testing, most reported being comfortable discussing HIV prevention with dentists; this supports the feasibility of HIV educational opportunities in dental settings. For patients who may have an inaccurate perception of their HIV risk, including those who report condomless sex with a partner of unknown HIV status or an HIV-positive partner who is not virally suppressed, patient-provider conversations would create opportunities for dentists or hygienists to correct misinformation about HIV prevention and acquisition.

Despite concerns that HIV screening in dental settings is not viable for “hard-to-reach” populations [16], our study provides evidence that HIV screening is acceptable and feasible when offering rapid tests in dental settings in areas that are disproportionately affected by HIV infection. Further, although it is common for underserved individuals and communities to experience challenges accessing dental or other healthcare, in some areas, community health centers are addressing the need. Testing in a dental setting may address key access or utilization barriers by leveraging existing community assets, such as a location frequented by a priority population and established, trusting relationships between dental professionals and patients that may not exist with other healthcare providers. Similarly, HIV testing in other nontraditional settings (e.g., community pharmacies, emergency rooms) may also offer testing opportunities for populations who may not regularly access traditional care settings.

Collaborations between public health and dental professionals can facilitate the successful expansion of HIV testing. For example, dental practices would need to be equipped at the individual and organizational level to implement rapid HIV testing [12]. Dental professionals may need training to enhance their knowledge about and comfort discussing HIV, in addition to how to administer oral rapid tests. Such trainings could be widely accessible if delivered in multiple venues (e.g., online, clinic in-service, conferences or professional meetings). Further, protocols that address privacy, providing support to patients after receipt of a positive test result and linking them to HIV care, and reimbursement for testing would be valuable tools for implementation [12,25,26]. Changes to dental school curricula would also be needed to equip future dental professionals [14,16]. Public health professionals could engage with the dental community and provide support in the development of educational materials [30] and protocols tailored to meet the needs of dental professionals in their respective settings. Public health professionals could also assist in framing messages for priority populations, whether communicated publicly or during patient-provider interactions. These messages can help convey accurate information about HIV risk and encourage testing without stigmatizing certain populations or geographic locations (e.g., using inflammatory or derogatory language). This is important because HIV stigma has the potential to decrease an individual’s likelihood of seeking HIV testing or care for fear of being ostracized [33], or to discourage a city or state from publicly addressing HIV prevention and care due to possible economic or other consequences (e.g., decreased tourism).

There were some limitations for our study. First, this research was conducted with the support of research staff in two FQHCs in South Florida. Consequently, it is unclear whether studies in other types of dental clinics (e.g., private practice), in other locations (e.g., rural areas, other metropolitan areas, states, or regions), or without research study staff aiding implementation would yield similar results. Second, although a recent qualitative study provided insight about the experiences of dental professionals who have offered HIV rapid testing [9], we did not conduct a process evaluation with the dental professionals’ in our study. Third, we were unable to examine linkage to HIV care because no one in our sample tested positive. However, the feasibility of successful linkage to care has been demonstrated [12,26]. Fourth, although the survey was administered via ACASI in a private room, some patients may not have felt comfortable disclosing information about their sexual behavior. Fifth, women comprised two-thirds of our sample. Although higher numbers of women utilize dental care [34], future studies should seek to enroll more men, as men are disproportionately affected by HIV infection. Lastly, there was no objective method to assess the accuracy of reports of partners’ HIV status. As a result, the data may have been subject to misclassification bias.

However, our study adds to the existing body of literature by examining HIV rapid testing uptake among patients in a high HIV prevalence area and elucidating reasons for accepting or declining testing. Although it is unlikely that routine, universal opt-out screening will be integrated into all dental settings, our findings provide support for the feasibility of offering testing in two FQHC settings that serve as a safety net for low-income persons who may have challenges accessing care. This study suggests certain practice aspects may enhance uptake, such as actively offering testing rather than using an intake form [27], collecting oral swabs near the beginning of the visit to address concern about lengthening one’s visit [27], and providing tests for free when possible [12,24,25]. Although a provider’s capacity to implement testing will vary based on infrastructure and available resources [26], even small-scale actions, such as discussions of HIV and referrals for HIV services in conjunction with other health-related discussions, could augment efforts to reach populations most vulnerable to HIV infection who may also have limited health care access.

### Public health implications

HIV testing plays a vital role in early detection and treatment for HIV infection. Integrating oral rapid HIV screening in dental settings offers an additional tool to provide access to persons who may be at risk for HIV exposure. These enhanced screening efforts that provide access to HIV testing in non-traditional settings could be advantageous. The existing body of research suggests most patients are willing to be tested in dental settings [23–27] and some providers and patients view oral rapid HIV testing as an extension of routine oral health care [18,20,25]. However, HIV screening in this venue has not yet gained traction [12]. Given that many patients may accept rapid HIV testing if offered during a dental visit, strengthening venue-based tools to decrease the potential missed opportunities in dental office settings is warranted and important as part of national HIV prevention strategies [35]. Members of the public health and dental community can work together to equip dental professionals to confidently communicate about HIV, conduct oral rapid HIV testing, and link HIV-positive individuals to care, especially in communities highly affected by HIV infection.

## Supporting information

S1 Appendix Patient acceptance of HIV rapid testing in the dental care setting (select questions from the exit interview). (DOCX)
