# Supplementary material for: Acceptability of oral rapid HIV testing at dental clinics in communities with high HIV prevalence in South Florida
Source: PLoS One. 2018 Apr 27;13(4):e0196323. doi: 10.1371/journal.pone.0196323 (PMC5922539; doi:10.1371/journal.pone.0196323)
Supplement: S1 Appendix — (DOCX) [file pone.0196323.s001.docx]

**Supporting Information**

**S1 Appendix** Patient Acceptance of HIV Rapid Testing in the Dental Care Setting (Select questions from the exit interview)

**HIV Testing History**

1. Other than today, have you ever been tested for HIV?

No

Yes

2. (If no) Why have you never been tested for HIV? (*Select all that apply)*

-Not enough time to take test and/or wait for results

-Confident he/she does not have a positive diagnosis and does not feel the test is necessary

-Does not feel comfortable taking the test

-Too embarrassed to take the test

-Fearful of the test results

-Does not want to discuss his/her sexual history

-Does not believe dental clinic is an appropriate setting to having testing performed

-Does not trust dentist/hygienist

-Fearful of the procedure

-Does not want to know the test result

-Does not feel that the test is important

-Concerned about his/her confidentiality

-Already undergoing HIV/AIDS treatment

3. Did you accept the rapid HIV test today?

No

Yes

4. (If yes) Why did you accept the test today? *(Select all that apply)*

- Patient thinks he/she is at risk

- Patient has never been tested before

- Patient's last HIV test was a long time ago

- The test is free

- Patient trusts the dentist/hygienist

- Patient believes that the dental clinic is an appropriate setting to having testing performed

- Patient wants to appease/reassure a partner and/or family member

- Patient suspects partner infidelity

- Patient will feel more comfortable knowing the result

5. What is the main reason you accepted the offer to be tested? (*Please mark only one answer*)

-It was important to me to know my result

-It was free

-I liked that I could have my result in 20 minutes

-I felt comfortable getting the test at the dentist instead of somewhere else

-Other reason: ___________________

6. (If no) Why did you refuse the test? *(Select all that apply)*

- Patient does not have enough time to take test and/or wait for the results

- Patient is confident that he/she does not have a positive diagnosis and does not feel the test is

necessary

- Patient does not feel comfortable taking the test

- Patient is too embarrassed to take the test

- Patient is fearful of the test results

- Patient does not want to discuss his/her sexual history

- Patient does not believe that the dental clinic is an appropriate setting to having testing

performed

- Patient does not trust dentist/hygienist

- Patient has already been tested recently

- Patient is already undergoing HIV/AIDS treatment

- Patient is fearful of the procedure

- Patient does not want to know the test result

- Patient does not feel that the test is important

- Patient is concerned about his/her confidentiality

- I am not at risk for HIV and don’t need a test

- I already know my HIV status

- I had an HIV test recently and don’t think I need another one

- I would be afraid to know I have HIV

- I would have to give my name

- I would be afraid of other people knowing my HIV test result

- I would never want to have the test at the dental clinic

- Getting a 20-minute HIV test would make my visit last too long

- I thought I would be in too much pain (toothache or other mouth pain) to have an HIV test

- I don’t think the 20-minute HIV test is correct
